# Glucose metabolism in the right middle temporal gyrus could be a potential biomarker for subjective cognitive decline: a study of a Han population

**DOI:** 10.1186/s13195-021-00811-w

**Published:** 2021-04-07

**Authors:** Qiu-Yue Dong, Tao-Ran Li, Xue-Yan Jiang, Xiao-Ni Wang, Ying Han, Jie-Hui Jiang

**Affiliations:** 1 Key laboratory of Specialty Fiber Optics and Optical Access Networks, Joint International Research Laboratory of Specialty Fiber Optics and Advanced Communication, School of Information and Communication Engineering, Shanghai University, Shanghai, China; 2 Department of Neurology, Xuanwu Hospital of Capital Medical University, Beijing, China; 3 German Center for Neurodegenerative Diseases, Clinical Research group, Venusberg Campus 1, Building 99, Bonn, Germany; 4 School of Biomedical Engineering, Hainan University, Haikou, China; 5 Center of Alzheimer’s Disease, Beijing Institute for Brain Disorders, Beijing, China; 6 National Clinical Research Center for Geriatric Disorders, Beijing, China

**Keywords:** Subjective cognitive decline, Alzheimer’s disease, FDG-PET, Glucose metabolic biomarker, Middle temporal gyrus

## Abstract

**Introduction:**

Subjective cognitive decline (SCD) represents a cognitively normal state but at an increased risk for developing Alzheimer’s disease (AD). Recognizing the glucose metabolic biomarkers of SCD could facilitate the location of areas with metabolic changes at an ultra-early stage. The objective of this study was to explore glucose metabolic biomarkers of SCD at the region of interest (ROI) level.

**Methods:**

This study was based on cohorts from two tertiary medical centers, and it was part of the SILCODE project (NCT03370744). Twenty-six normal control (NC) cases and 32 SCD cases were in cohort 1; 36 NCs, 23 cases of SCD, 32 cases of amnestic mild cognitive impairment (aMCIs), 32 cases of AD dementia (ADDs), and 22 cases of dementia with Lewy bodies (DLBs) were in cohort 2. Each subject underwent [18F]fluoro-2-deoxyglucose positron emission tomography (PET) imaging and magnetic resonance imaging (MRI), and subjects from cohort 1 additionally underwent amyloid-PET scanning. The ROI analysis was based on the Anatomical Automatic Labeling (AAL) template; multiple permutation tests and repeated cross-validations were conducted to determine the metabolic differences between NC and SCD cases. In addition, receiver operating characteristic curves were used to evaluate the capabilities of potential glucose metabolic biomarkers in distinguishing different groups. Pearson correlation analysis was also performed to explore the correlation between glucose metabolic biomarkers and neuropsychological scales or amyloid deposition.

**Results:**

Only the right middle temporal gyrus (RMTG) passed the methodological verification, and its metabolic levels were correlated with the degrees of complaints (*R* = − 0.239, *p* = 0.009), depression (*R* = − 0.200, *p* = 0.030), and abilities of delayed memory (*R* = 0.207, *p* = 0.025), and were weakly correlated with cortical amyloid deposition (*R* = − 0.246, *p* = 0.066). Furthermore, RMTG metabolism gradually decreased across the cognitive continuum, and its diagnostic efficiency was comparable (NC vs. ADD, aMCI, or DLB) or even superior (NC vs. SCD) to that of the metabolism of the posterior cingulate cortex or precuneus.

**Conclusions:**

These findings suggest that the hypometabolism of RMTG could be a typical feature of SCD, and the large-scale hypometabolism in patients with symptomatic stages of AD may start from the RMTG, which gradually progresses starting in the preclinical stage. The specificity of identifying SCD from the perspective of self-perceived symptoms is likely to be increased by the detection of RMTG metabolism.

**Supplementary Information:**

The online version contains supplementary material available at 10.1186/s13195-021-00811-w.

## Introduction

Alzheimer’s disease (AD) represents the leading cause of dementia, which is a distinct pathological entity that locks into a long clinical latency and lacks disease-modifying therapy [1]. Over the past decades, a string of disappointing clinical trial results has forced researchers to shift our focus to the preclinical stage of AD, which represents the most promising therapeutic window [1, 2]. Subjective cognitive decline (SCD) is a state in which subjects have self-experienced persistent cognitive decline in the absence of objective impairment [3]. In comparison to normal control (NC) subjects, increasing amounts of evidence suggest that SCD subjects are at an increased risk for developing future objective cognitive decline [4–6]. As a representation of the preclinical stage of AD [3], the accurate detection of AD-sourced SCD is of great importance for disease prediction, early disease screening, early interventions, and even drug development. However, the current diagnosis of SCD is largely based on self-perceived symptoms, and the specificity of identifying subjects at risk of future cognitive deterioration is very low. Research on brain functional changes and imaging biomarkers for these subjects is still in its infancy, and there is an urgent need for objective indicators to improve the specificity of the diagnosis.

Glucose metabolism is an important physiological index for indicating the function of brain neurons. It has been integrated as a neurodegenerative biomarker by the latest National Institute on Aging-Alzheimer’s Association (NIA-AA) diagnostic framework of AD [7]. Previous studies have proposed an AD-related metabolic pattern by using the [18F]fluoro-2-deoxyglucose (FDG) positron emission tomography (PET) imaging technique, which is characterized by the hypometabolism of several regions of interest (ROIs), including the default mode network, parieto-temporal association areas, posterior cingulate cortex (PCC), precuneus (PCUN), etc. [8, 9]. Based on the above, researchers further explored the brain glucose metabolic abnormalities in SCD and proposed that hypometabolism probably began to appear in certain regions and finally spread throughout the entire AD-related metabolic pattern with gradual damage to the neurons [10, 11]. More specifically, Scheef et al. reported that the PCUN was a robust hypometabolic region of SCD [12], but this result was not supported by a previous study [13], which found several scattered areas mainly in the parieto-temporal association areas and medial temporal lobe. Thus, it is still under debate which ROI could be the most accurate biomarker for glucose metabolism in SCD populations.

The objective of this study was to explore the glucose metabolic biomarker of SCD at the ROI level in the hopes of predicting which SCD subjects are more likely to develop cognitive deterioration in the future. FDG-PET images were used to detect glucose metabolism in brains in vivo. SCD subjects from Han populations were included in this study to exclude the possible influence of different cultural backgrounds [14, 15]. To reduce the influence of data selection, the subjects in our study were from two tertiary medical centers, and we performed permutation tests and repeated cross-validations to improve the robustness of the results. In addition, participants with cognitive impairment were also recruited to explore the changes in metabolic biomarkers across the cognitive continuum.

## Materials and methods

### Participants

The participants in this study were from the Sino Longitudinal Study on Cognitive Decline (SILCODE) project. The SILCODE project is a registered ongoing multicenter AD study on the community Han population of mainland China (ClinicalTrials.gov identifier: NCT03370744; the protocol can be accessed at ClinicalTrials.gov) [16]. In this study, 62 NC individuals and 55 individuals with SCD were enrolled. To verify the effectiveness of glucose metabolic biomarkers across the whole dementia disease spectrum, we also selected 32 patients with amnestic mild cognitive impairment (amnestic MCI [aMCI]), 32 with AD dementia (ADD), and 22 with dementia with Lewy bodies (DLB) from the SILCODE project. Magnetic resonance imaging (MRI) and FDG-PET images were selected for all subjects. Notably, among these participants, 26 NC and 32 SCD subjects were selected from the Tiantan Hospital (Capital Medical University; a subcenter of SILCODE project), and they additionally had amyloid-PET (Florbetapir F-18 [AV45]) images taken, in which 38.5% of the NC (*n* = 10) and 37.5% of the SCD subjects (*n* = 12) were classified as amyloid positivity according to a priori with the established cutoff of cortical standardized uptake value ratio (SUVR) > 1.18 [17, 18]; the remaining subjects were all recruited from Xuanwu Hospital of Capital Medical University (center of SILCODE project), and they did not undergo amyloid-PET scans.

We divided these participants into two cohorts: the subjects from Tiantan Hospital were defined as cohort 1 (*n* = 58; NC1 and SCD1), and those from Xuanwu Hospital were defined as cohort 2 (*n* = 145; NC2, SCD2, aMCI, ADD, and DLB). There was no intersection between cohorts 1 and 2 in the dataset.

In addition to the demographic data and apolipoprotein E (APOE) genotype, the NC (NC1 + NC2) and SCD (SCD1 + SCD2) subjects had a detailed examination for cognitive function, including the Mini-Mental State Examination (MMSE) and the Montreal Cognitive Assessment-Basic (MoCA-B) Chinese Version scales for global cognition, the Subjective Cognitive Decline-9 (SCD-9) scale for subjective complains, the Auditory verbal learning test-long delayed memory (AVLT-N5) and recognition (AVLT-N7) scales for memory domain, the Hamilton Depression (HAMD) and Hamilton Anxiety (HAMA) scales for emotions, and others [16]. The participants with aMCI also had demographic data collected and underwent detailed neuropsychologic examinations, while the participants with dementia only had demographic data collected and MMSE tests administered.

### Inclusion criterion

All participants were right-handed and Mandarin-speaking. The NC participants were volunteers without any concerns about cognitive decline and whose neuropsychologic test scores were in the normal range. The entry criterion of SCD referred to the conceptual framework proposed by Jessen et al. in 2014 [19] and our previous references [16, 20], including (a) self-reported experience of persistent decline in memory compared to a previous state (within the last 5 years), (b) persistent concerns about memory changes, and (c) performance within the normal range on all clinical scales (adjusted for age, sex, and education; versions suitable for the Chinese). The diagnosis of aMCI was based on a neuropsychological method [21]. ADD refers to the guidelines from NIA-AA workgroups [7, 22] and DLB is based on a previous criterion [23]. Participants were excluded if they had a history of stroke, brain damage, severe anemia, syphilis infection, or other conditions [16]. The diagnoses were checked by two experienced neurologists (Ying Han and Tao-Ran Li).

All participants, or their informants/caregivers, provided written informed consent and written consent to permit the publication of their anonymized clinical details. A flowchart and further details regarding the evaluation of our participants are presented in supplementary Figure 1.

### Imaging acquisition protocol

Participants from the two cohorts were all scanned with the same machine model and parameters. The MRI and PET images were both acquired with a simultaneous hybrid PET/MR scanner (SIGNA; GE Healthcare, Chicago, IL, USA). Before undergoing imaging, the subjects were instructed to keep their eyes closed but not to fall asleep, to relax their minds, and to move as little as possible during imaging; foam pads and headphones were used to minimize head movement and imager noise.

T1-weighted images were acquired with a magnetization-prepared rapid gradient echo sequence: field of view (FOV) = 256 × 256 mm^2^, matrix = 256 × 256, slice thickness = 1 mm, gap = 0, slice number = 192, repetition time (TR) = 6.9 ms, echo time (TE) = 2.98 ms, inversion time = 450 ms, flip angle = 12°, voxel size = 1 × 1 × 1 mm^3^. For FDG-PET, subjects were fasted for at least 6 h, and their blood glucose level was < 120 mg/dL. The images were acquired 40 min after intravenous injection of [18F]-FDG (3.7 MBq/kg), and the data were recorded by using a time-of-flight ordered subset expectation maximization algorithm with the following parameters: scan duration = 35 min, eight iterations, 32 subsets matrix = 192 × 192, FOV = 350 × 350, half-width height = 3. The imaging acquisition protocol of amyloid-PET was basically the same as FDG-PET, with the tracer Florbetapir F-18 of 7–10 mCi. Notably, the interval time between the two PET scans was at least 3 days to eliminate the effects of the first tracer.

### PET imaging preprocessing

First, the all original DICOM PET and T1-weighted images were converted to the NIfTI file format using DCM2NII (https://people.cas.sc.edu/rorden/mricron/dcm2nii.html). Second, the gray matter (GM), white matter, and cerebrospinal fluid images were segmented from the T1-weighted images by using the CAT12 toolbox (http://dbm.neuro.uni-jena.de/cat/). Third, the PET images were coregistered with their corresponding T1-weighted images and then corrected for partial volume effects (PVE) based on the Muller-Gartner algorithm [24]. Fourth, the GM images were normalized to the standard Montreal Neurological Institute (MNI) space by using the Statistical Parametric Mapping (SPM8; https://www.fil.ion.ucl.ac.uk/spm/software/spm8); subsequently, the PVE-corrected PET images were normalized to the MNI standard space using the forward transformation parameters determined by T1-weighted image spatial normalization. Finally, these PET images were smoothed with an 8-mm full width at half maximum Gaussian kernel.

For FDG-PET, the smoothed images for each subject were normalized to obtain the SUVR map using the cerebral cortex as a reference region. For amyloid-PET, the whole cerebellum was used as the reference region, and the whole cerebral cortex was used as the ROI [18].

### ROI analysis

The ROI analysis was based on the Anatomical Automatic Labeling (AAL) template [25], aiming to explore glucose metabolic biomarkers for SCD. First, we calculated the SUVR of each ROI (90 regions) in the NC1 and SCD1 groups, with age, sex, and education as covariates, and we made group comparisons for each ROI between the two groups. Second, to verify the repeatability of the above biomarkers, data from cohort 2 (NC2 and SCD2) were used as an external validation dataset. The covariate-adjusted SUVR was calculated for all ROIs, and group comparisons were also tested. Only those ROIs that had statistically significant differences in both cohorts were considered potential glucose metabolic biomarkers for SCD.

### Voxel analysis

In parallel, to verify the results of the ROI analysis, an independent two-sample t-test at the voxel level in the whole cerebral cortex was performed on the complete individual datasets of the NC and SCD groups under regression of sex, age and education. The GM probability map was included as a voxel-based covariate to address the variability in the GM density in the different populations due to the effect of GM atrophy on these comparisons.

### Statistical analysis

In this study, scalar statistical analysis was conducted using IBM SPSS statistics v25.0 (SPSS Inc., Chicago, USA), and the statistical significance level was set at *p* < 0.05. The voxel-based statistical evaluation was performed in Data Processing & Analysis for Brain Imaging (DPABI, http://rfmri.org/dpabi).

#### Demography and neuropsychology

The demographic and neuropsychological data were summarized as numbers (%) or means ± standard deviations for categorical and continuous variables, respectively. The group comparisons of categorical variables were performed by using the chi-square test for sex and APOE status. The two-sample t-test was performed between NC1 and SCD1 from cohort 1 for all continuous variables and between NC2 and SCD2 from cohort 2 for all continuous variables except age, education, and MMSE; one-way analysis of variance (ANOVA) followed by post hoc test was performed among the five groups (NC2, SCD2, aMCI, ADD, DLB) for age, education, and MMSE.

#### The identification and validation of SCD glucose metabolic biomarkers

For ROI analysis, to identify the SCD glucose metabolic biomarkers, 1000 permutation tests were performed in cohorts 1 and 2, identically and independently. Furthermore, to avoid the influence of sample selection and to enhance the robustness of the results, this study performed twofold cross-validations for NC and SCD (cohorts 1 and 2) with 10 repeats, and 1000 permutation tests were also performed in both subdatasets.

For voxel analysis, the Gaussian random field (GRF) correction (voxel level *p* < 0.01, cluster level *p* < 0.05) was applied to each t-map, followed by an observation of group differences in the spatial distribution.

To explore the changes in the cognitive continuum of the SCD glucose metabolic biomarkers, the covariates adjusted biomarkers in the aMCI, ADD, and DLB groups were also calculated. One-way ANOVA and post hoc tests (Bonferroni correction) were used to compare the differences of the biomarkers among the NC2, SCD2, aMCI, ADD, DLB groups in cohort 2 and among the NC, SCD, aMCI, ADD, and DLB groups in cohort 1 combined with cohort 2.

#### ROC analysis

Receiver operating characteristic (ROC) curves were used to evaluate the capabilities of the potential glucose metabolic biomarker in distinguishing different groups: NC1 vs. SCD1 from cohort 1; NC2 vs. SCD2 from cohort 2; NC vs. SCD from cohort 1 combined with cohort 2; and NC2 vs. aMCI or ADD or DLB from cohort 2. The areas under curves (AUCs) with 95% confidence intervals (CIs) were calculated. We also compared the corresponding ROC curves of typical areas of AD. The PCC and PCUN were selected, both of which were manually drawn on the study-specific template.

#### Correlation analysis

To explore whether correlations exist between the levels of SCD glucose metabolic biomarkers and the neuropsychological scales (NC and SCD) as well as amyloid deposition (NC1 and SCD1), Pearson correlation coefficients were calculated; correlation analysis was also performed in regions of the PCC and PCUN.

## Results

### Background characteristics

The detailed demographic and clinical characteristics of both cohorts are presented in Table 1. In cohort 1, compared with the NC1 group, the SCD1 group showed a higher proportion of women (*p* = 0.025) and a higher score on the SCD-9 scale (*p* < 0.001). In cohort 2 (NC2, SCD2, aMCI, ADD, DLB), the sex was unbalanced among the groups (*p* < 0.001); specifically, the SCD2 group was dominated by women, while the DLB group was mainly men. Besides, the average age of the ADD group was significantly lower than the others (*p* < 0.001), while for the MMSE scale, the average score gradually decreased across the cognitive continuum, with the highest in the NC2 and SCD2 groups, a decrease in the aMCI group, and the lowest in the ADD and DLB groups. In addition, the SCD2 group showed higher scores on the SCD-9 scale (*p* < 0.001), HAMD scale (*p* = 0.002), and HAMA scale (*p* = 0.006) than the NC2 group.

**Table 1:** Clinical characteristics of the participants

	Cohort 1 (***n*** = 58)		Cohort 2 (***n*** = 145)				
	NC1 (26)	SCD1 (32)	NC2 (36)	SCD2 (23)	aMCI (32)	ADD (32)	DLB (22)
**Female,** ***n*** **(%)**	13 (50.0%)	25 (78.1%)^*^	23 (63.9%)	21 (91.3%)	14(43.8%)	13(40.6%)	1(4.5%)^††^
**Age**	66.38 ± 4.51	66.00 ± 4.83	65.16 ± 4.26	65.82 ± 4.28	67.81 ± 6.99	60.66 ± 8.87	66.91 ± 8.41^††^
**Education**	13.42 ± 2.88	13.18 ± 2.64	12.68 ± 2.86	13.04 ± 2.73	13.03 ± 3.02	13.10 ± 4.15	13.05 ± 3.65
**APOE ε4,** ***n*** **(%)**	9 (34.6%)	7 (21.9%)	7 (19.4%)	6 (26.1%)	N/A	N/A	N/A
**MMSE**	29.34 ± 0.79	29.12 ± 0.90	29.22 ± 0.92	29.00 ± 0.67	25.53 ± 3.45	19.09 ± 5.77	20.05 ± 4.99^††^
**MoCA-B**	26.00 ± 1.87	26.75 ± 1.70	26.63 ± 2.05	26.04 ± 1.63	22.66 ± 1.73	N/A	N/A
**SCD-9**	2.98 ± 2.25	5.29 ± 1.92^**^	3.23 ± 1.79	5.06 ± 1.50^**^	5.59 ± 1.32	N/A	N/A
**HAMD**	2.34 ± 2.63	3.37 ± 3.12	1.41 ± 1.66	4.00 ± 3.95^*^	2.38 ± 1.52	N/A	N/A
**HAMA**	2.96 ± 2.44	4.46 ± 3.27	2.91 ± 2.83	5.34 ± 3.74^*^	2.56 ± 1.68	N/A	N/A
**AVLT-N5**	7.67 ± 1.89	7.34 ± 1.67	7.65 ± 2.03	7.81 ± 1.87	4.01 ± 1.67	N/A	N/A
**AVLT-N7**	22.46 ± 1.42	22.65 ± 1.38	22.50 ± 1.44	22.52 ± 1.75	16.73 ± 2.58	N/A	N/A

Categorical and continuous measures are presented as numbers (%) or as means ± standard deviations. Statistical analyses were conducted by chi-square tests for categorical variables, independent two-sample two-tailed t-tests (MoCA-B, SCD-9, HAMD, HAMA, AVLT-N5, and AVLT-N7 in cohorts 1 and 2) or one-way ANOVA followed by post hoc tests (education, MMSE in cohort 2) for continuous variables. Comparisons between the two groups, * means *p* < 0.05, ** means *p* < 0.001; comparisons among the five groups, †† means *p* < 0.001, results of the post hoc tests were not marked; the neuropsychological scales of MoCA-B, SCD-9, HAMD, HAMA, AVLT-N5, and AVLT-N7 were not compared among the three groups of NC2, SCD2, and aMCI

*Abbreviations*: *NC* normal control, *SCD* subjective cognitive decline, *aMCI* amnestic mild cognitive impairment, *ADD* AD-dementia, *DLB* dementia with Lewy body, *APOE* apolipoprotein E, *MMSE* Mini-Mental State Examination, *MoCA-B* Montreal cognitive assessment-basic, *SCD-9* Subjective Cognitive Decline-9, *HAMD* Hamilton depression scale, *HAMA* Hamilton anxiety scale, *AVLT-N5* Auditory verbal learning test-long delayed memory, *AVLT-N7* Auditory verbal learning test-recognition, *ANOVA* analysis of variance, *N/A* not applicable

Notably, there were no differences between NC1 and NC2 or between SCD1 and SCD2.

### RMTG hypometabolism as the SCD glucose metabolic biomarker

#### ROI analysis

**Fig. 1:**
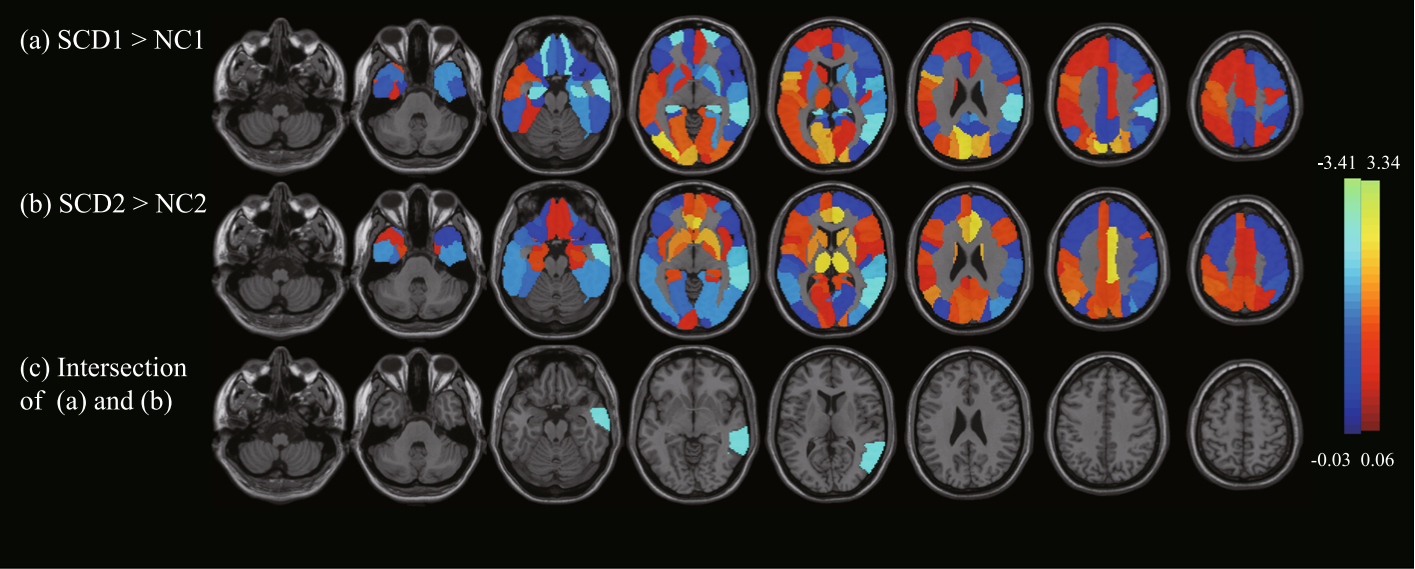
The results of SCD glucose metabolic biomarkers based on ROI analysis. In the metabolic comparisons between SCD patients and NCs, this study considered the 90 regions (AAL template) as ROIs and calculated the mean SUVR value of each ROI, which was adjusted for age, sex, and education. Permutation tests 1000 times were used to find significant differences between NC1 and SCD1 as well as between NC2 and SCD2. **a** and **b** show the SCD regional changes of 90 ROIs compared with NC, where **a** NC1 and SCD1 were used from cohort 1, **b** NC2 and SCD2 were used from cohort 2, and **c** shows the intersection areas of significantly different regions in (**a**) and (**b**). The regions with metabolic changes of SCD are overlaid on the structural MRI template images. Cool colors represent voxels with negative region weights and hypometabolism, and hot colors represent voxels with positive weights and hypermetabolism. Abbreviations: SCD, subjective cognitive decline; ROI, region of interest; NC, normal control; AAL, anatomical automatic labeling; SUVR, standardized uptake value ratio; MRI, magnetic resonance imaging

Figure 1 shows the metabolic changes of SCD compared with NC of the 90 ROIs. Figure 1a shows the metabolic changes of the SCD individuals in cohort 1. SCD was significantly hypometabolic in the bilateral superior orbital frontal gyrus and hippocampus, right rolandic operculum, supramarginal gyrus and middle temporal gyrus (MTG), and hypermetabolic in the bilateral calcarine, cuneus, and superior occipital gyrus, left inferior opercular frontal gyrus and rolandic operculum, and right inferior occipital gyrus. Figure 1b shows the metabolic changes of SCD individuals in cohort 2. The SCDs were significantly hypometabolic in the bilateral inferior occipital gyrus lingual gyrus and MTG, right middle occipital gyrus, fusiform gyrus, superior temporal gyrus, and left inferior temporal gyrus and were hypermetabolic in the bilateral anterior cingulate gyrus, paracingulate gyrus, left angular gyrus, right median cingulate gyrus, and paracingulate gyrus. Figure 1c shows the intersection area of the significantly altered area of the two cohorts; only the right MTG (RMTG) was retained in both cohorts 1 (*p* < 0.05) and 2 (*p* < 0.001). The RMTG was the only brain area that had significant differences in each subset of the 10 repeated two-fold cross-validations; the details are listed in supplementary Table 1.

#### Voxel analysis

The results of voxel analysis found that the SCD individuals had hypometabolism in the temporal lobe, supplementary motor area, angular, lingual, fusiform, and partial orbital frontal lobe and hypermetabolism in the partial occipital lobe and frontal lobe (GRF uncorrected; *p* < 0.05, cluster size > 100) when compared with NCs. Table 2 shows the detailed regions in the AAL atlas based on the voxel analysis. Notably, the results of the ROI analysis and voxel analysis both found hypometabolism of RMTG; thus, RMTG hypometabolism was identified as a potential glucose metabolic biomarker of SCD in the Han population.

**Table 2:** Brain regions with significant differences between SCD and NC based on voxel analysis

MNI coordinate (mm)			Cluster location (AAL template)	Hemisphere	Cluster size	Metabolic change from SCD to NC
*X*	*Y*	*Z*				
50	− 60	24	Temporal_Mid; Angular; Temporal_Sup	Right	573	Hypometabolism
60	− 26	16	Temporal_Sup; SupraMarginal; Postcentral; Precentral	Right	492	Hypometabolism
30	− 46	− 42	Fusiform; ParaHippocampal; Temporal_Pole_Mid; Temporal_Inf	Right	332	Hypometabolism
54	− 40	34	SupraMarginal; Parietal_Inf; Temporal_Sup; Temporal_Mid	Right	297	Hypometabolism
− 24	54	− 12	Frontal_Sup_Orb; Frontal_Mid_Orb; Rectus; Frontal_Med_Orb	Left	262	Hypometabolism
− 40	8	− 38	Temporal_Inf; Temporal_Pole_Mid; Temporal_Pole_Sup; Fusiform	Left	257	Hypometabolism
24	− 48	− 6	Lingual; Fusiform	Right	216	Hypometabolism
18	− 36	− 20	Frontal_Sup_Orb; Frontal_Mid_Orb; Frontal_Med_Orb	Right	188	Hypometabolism
− 20	− 14	− 14	Hippocampus; Amygdala;	Left	162	Hypometabolism
52	− 4	− 34	Temporal_Inf; Temporal_Mid	Right	153	Hypometabolism
62	− 10	−18	Temporal_Mid; Temporal_Sup	Right	146	Hypometabolism
− 62	− 14	− 14	Temporal_Mid	Left	144	Hypometabolism
4	− 94	8	Calcarine; Cuneus; Occipital_Mid	Left, right	505, 166	Hypermetabolism
− 20	− 86	34	Occipital_Sup; Cuneus; Occipital_Mid	Left	371	Hypermetabolism
− 20	− 74	52	Parietal_Sup; Precuneus; Parietal_Inf	Left	210	Hypermetabolism
4	24	44	Frontal_Sup_Medial; Supp_Motor_Area	Left, right	90, 111	Hypermetabolism
28	52	− 10	Frontal_Mid_Orb; Frontal_Inf_Orb	Right	159	Hypermetabolism
44	16	2	Insula; Frontal_Inf_Oper	Right	153	Hypermetabolism
− 4	50	16	Frontal_Sup_Medial; Cingulum_Ant	Left	138	Hypermetabolism
4	− 62	66	Precuneus	Left; right	64, 52	Hypermetabolism
18	− 68	58	Parietal_Sup	Right	114	Hypermetabolism

Voxel analysis between NC (NC1 + NC2) and SCD (SCD1 + SCD2). The threshold of the t-map was set to *p* < 0.05, cluster size > 100

*Abbreviations*: *MNI* Montreal Neurological Institute, *AAL* anatomical automatic labeling, *SCD* subjective cognitive decline, *NC* normal control, *mid* middle, *sup* superior, *inf* inferior, *orb* orbital, *ant* anterior

### SCD glucose metabolic biomarkers across the cognitive continuum

**Fig. 2:**
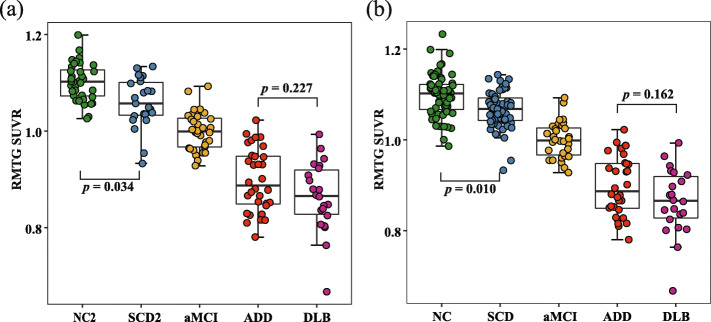
The metabolism of RMTG in the cognitive continuum. **a** Plot showing RMTG SUVR of NC2, SCD2, aMCI, ADD, and DLB; **b** plot showing RMTG SUVR of NC, SCD, aMCI, ADD, and DLB. The SUVR between SCD2 and NC2 (*p* = 0.034) as well as SCD and NC (*p* = 0.010) both had significant differences; there were no differences between the ADD and DLB groups (*p* = 0.227 in a, *p* = 0.162 in **b**) but there were significant differences for the remaining combinations (*p* < 0.001 both in **a** and **b**; not marked in Fig. 2). The above *p* values were all subjected to Bonferroni correction. Abbreviations: RMTG, right middle temporal gyrus; SUVR, standardized uptake value ratio; NC, normal control; SCD, subjective cognitive decline; aMCI, amnestic mild cognitive impairment; ADD, AD-dementia; DLB, dementia with Lewy body

To visually present the dynamic changes in the metabolic level of RMTG across the cognitive continuum, five typical cases from different groups (NC, SCD, aMCI, ADD, and DLB) were selected; supplementary Figure 2 shows their single-subject RMTG SUVR maps, suggesting that with the deterioration of cognition, metabolism may gradually decrease. Furthermore, Fig. 2a shows the results of group comparisons for RMTG SUVR among the NC2, SCD2, aMCI, ADD, and DLB groups. The post hoc analysis results showed that there was a significant metabolic difference between SCD2 and NC2 (*p* = 0.034), and there were also significant group differences between the remaining groups (*p* < 0.001), except for the comparison between ADD and DLB (*p* = 0.227). As shown in Fig. 2b, the differences between groups still existed when participants in the two cohorts were mixed. The *p* value was 0.010 between the NC and SCD groups, 0.162 between the ADD and DLB groups, and less than 0.001 for the remaining combinations.

### ROC analysis

**Fig. 3:**
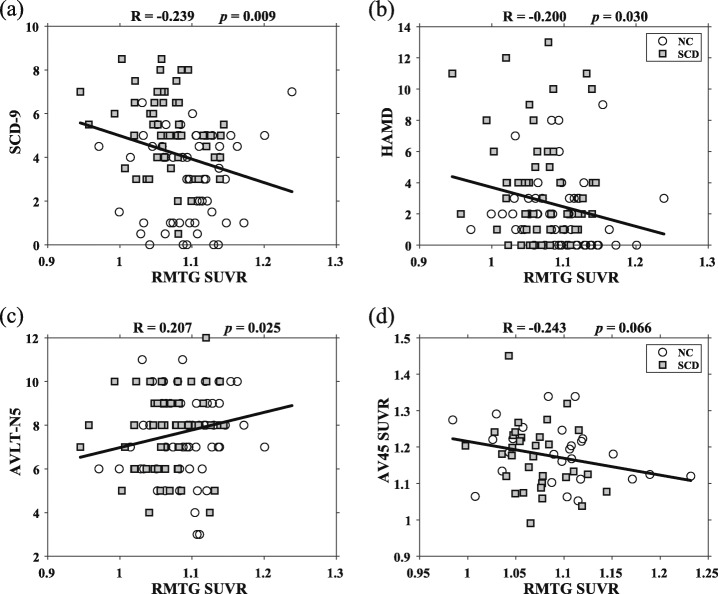
The results of correlation analysis. The metabolism of RMTG showed correlations with the scores of SCD-9 (**a**), HAMD (**b**), AVLT-N5 (**c**), and AV45 SUVR (**d**). More complaints and depression were related to a decreased glucose metabolism of RMTG. AVLT-N5 was positively correlated with RMTG SUVR. The more Aβ deposition, the lower the RMTG metabolism, but it did not reach a significant difference level. Hollow circles indicate NC individuals, solid squares mean SCD individuals, the solid line is the fitted line. Abbreviations: NC, normal control; SCD, subjective cognitive decline; RMTG, right middle temporal gyrus; SUVR, standardized uptake value ratio; SCD-9, Subjective Cognitive Decline-9; HAMD, Hamilton depression scale; AVLT-N5, auditory verbal learning test-long delayed memory; AV45, Florbetapir F-18

In distinguishing NC from the other groups, the abilities of three indicators, the metabolism of RMTG, PCC, and PCUN, were compared by using ROC analysis. As shown in the supplementary material (Fig. 3 and Table 2), RMTG achieved the largest AUCs in distinguishing NC from SCD (NC1 vs. SCD1, NC2 vs. SCD2, and NC vs. SCD; 0.638–0.717) when compared with the PCC (0.534–0.604) or the PCUN (0.499–0.562), and in distinguishing NC from the cognitively impaired groups (aMCI, ADD, and DLB), the AUCs of RMTG (0.959–1.000) were also comparable or even larger than the PCC (0.899–0.935) or the PCUN (0.617–0.741). Detailed information, such as the 95% CIs, are presented in the supplementary material.

### Correlation analysis

Figure 3 shows the correlations between RMTG metabolism and the neuropsychological scores. The results showed that RMTG metabolism was significantly correlated with the SCD-9, HAMD, and AVLT-N5 scales, and there were no correlations with the other scales. The metabolism of PCC and PCUN had no correlations with the neuropsychological scales. Specifically, RMTG metabolism was negatively correlated with SCD-9 (*R* = − 0.239, *p* = 0.009, Fig. 3a) and HAMD (*R* = − 0.200, *p* = 0.030, Fig. 3b) and positively correlated with AVLT-N5 (*R* = 0.207, *p* = 0.025, Fig. 3c), indicating that serious complaints and depression, as well as a poor performance on the delayed recall test, were all correlated with decreased metabolic levels of RMTG. In addition, although we found a negative correlation between RMTG metabolism and cortical amyloid deposition, the correlation did not reach the level of statistical significance (*R* = − 0.246, *p* = 0.066, Fig. 3d).

## Discussion

In this study, we found that glucose hypometabolism of RMTG is a robust biomarker of SCD. More specifically, this hypometabolic region was identified in both ROI and voxel-based analyses and verified by using a method combining multiple permutation tests and repeated cross-validations from two independent cohorts from two tertiary medical centers. Furthermore, RMTG metabolism was gradually decreased across the cognitive continuum and showed a better classification ability in distinguishing NC from individuals with other cognitive stages than typical hypometabolic regions of AD, including the PCC and PCUN. In addition, the biomarker showed significant correlations with degrees of subjective complaints, depressions, and abilities of delayed memory. Our results indicate that individuals with SCD have already developed limited changes in the brain metabolism, and the hypometabolic regions are likely to spread gradually accompanied by a deterioration of cognition. The accurate identification of AD-sourced SCD theoretically provides a therapeutic window earlier than MCI [3], and the specificity in identifying SCD from the perspective of self-perceived symptoms is likely increased by the detection of RMTG metabolism in these individuals.

Recent studies have largely focused on identifying the qualitative and quantitative features of SCD that are specifically related to the underlying AD pathology [26]; in contrast, previous contradictory results of FDG-PET in this group have been somewhat neglected. ROI-based glucose metabolic biomarkers are easily extracted and quantified and may have clinical meaning, and this method has also been frequently used in AD research [27–29]. Several research groups tried to explore the ROI-based metabolic pattern of SCD, and the results showed that its metabolism was similar to that of NC [30, 31] and not influenced by Aβ deposition [32]. Other studies performed voxel-based analyses, suggesting that individuals with SCD had a significant reduction in glucose metabolism in the periventricular regions [33] or other scattered areas [13] or had no metabolic changes [34, 35] when compared with NCs. Importantly, one study performed both ROI-based and voxel-based analyses, and the results showed that hypometabolism of the right precuneus is a typical feature of SCD [12]. The large intra-group differences among SCD samples and mismatches between groups may explain some of the inconsistent results; for example, the age span of SCD subjects in one study reached 31 years, from 53 to 84 years old [34], and in another study, the presence of the APOE ε4 allele in the SCD subjects was 0%, while it was 52% in the NCs [30]. In addition, it should be noted that the ROIs selected in previous studies were all based on prior knowledge, which may be affected by different samples, and cannot reflect the characteristics of the preclinical state; a whole brain study without hypotheses can avoid this problem to some extent and help detect early limited functional changes. In our study, we have included data from two centers, and all data strictly followed the inclusion criteria of our project. We performed methodological optimizations to increase the robustness of the results and to settle previous disputes. First, we combined undifferentiated cortical ROI-based analysis and voxel-based analysis. Second, multiple permutation tests and test-retest methods of data from the two centers were performed. Third, the selected hypometabolic region was further verified by repeated cross-validations. Therefore, we thought the glucose hypometabolism of RMTG reflected by FDG-PET is likely to be a reliable biomarker of SCD in methodology and be a good additional index for the inclusion of SCD since it can reduce the individual errors caused by subjective descriptions to a certain extent.

Previous studies have not reported the metabolic changes of RMTG in individuals with SCD. This novel finding may provide a new perspective for the disease changes of SCD or the spectrum of AD. According to previous reports, the MTG region has close functional connectivity with the hippocampus [36], is primarily involved in verbal or semantic cognition, and is also associated with oral memory [37]; furthermore, it represents a signature area of cortical atrophy in patients with symptomatic stages of AD [38–41]. These results indicate the important roles of MTG in the Alzheimer’s continuum. Furthermore, the RMTG in the AAL template is an important part of the default mode network [42], and a study performed by Lim et al. previously showed that the RMTG has already developed slight atrophy as early as the SCD stage [43], providing a structural basis for our results. Specifically, we supposed that structural atrophy may be due to the death of neurons and then lead to a decrease in metabolism. Thus, it seems reasonable that the hypometabolic region of SCD is located in the RMTG. In our study, the metabolic difference of MTG was only observed on the right side, which is consistent with the atrophy side of MTG in the SCD subjects [43] and is also consistent with the side of the MTG in the default network [42]; however, the specific reason for this is still unclear currently, which may be related to the laterality, and there may be some unexplained disease-related mechanisms in the right hemisphere. Previous studies have found that the function and atrophy patterns of the bilateral temporal lobes were asymmetric in patients with neurodegenerative diseases [44–48], and cerebral glucose metabolism in the bilateral hemispheres was also significantly different in healthy individuals [49]. To further verify the clinical rationality, individuals with other stages on the cognitive continuum, other than NC and SCD, were also enrolled to make cross-sectional comparisons. Although the average age of patients in the ADD group was lower than that in the other groups, the metabolism of RMTG was still gradually decreased across the cognitive continuum, suggesting that the decrease in metabolism might be due to cognitive changes instead of the influences of aging, and the RMTG was damaged as early as the SCD stage and the damage gradually progressed, accompanied by cognitive deterioration. Importantly, compared with the PCC and PCUN, which are thought to be characteristic hypometabolic regions of AD [8–10, 50], the ROC analysis showed that the metabolism of RMTG was better in distinguishing NC from SCD, as well as NC from symptomatic patients. These results support our hypothesis to some extent that the hypometabolic abnormalities in the dementia stage may start from a local area and then gradually spread into signature regions. In other words, the RMTG may be the seed region. Considering the heterogeneity of SCD, we also included patients with DLB; since ADD and DLB are similar in metabolic patterns [51], it is understandable that there was no metabolic difference in the RMTG between them. We noted that the ability of RMTG to distinguish NC from SCD was not outstanding (AUC = 0.638–0.717), but we thought it was within a reasonable range when compared with previous studies of monoparametric MRI [52–54]. For example, Peter et al. proposed a multivariate pattern recognition framework integrating the gray matter atrophy pattern in the differentiation of SCD from NC, and the AUC was 0.67 [52]. The studies performed by our group suggested that the classification performance was approximately 60% for diffusion tensor imaging [54] and approximately 70% for functional MRI [53, 54]. From another perspective, we found that the glucose metabolism of RMTG was correlated with the abilities of delayed memory, which was consistent with previous reports [35, 55, 56]. Other studies have suggested relationships between the structure and function of this area and emotions [57, 58], which also confirms our results that the metabolism of RMTG is related to depression. We also observed that the degree of complaints was negatively correlated with metabolism, which was supported by a recent study where the degree of self-reported SCD was negatively correlated with glucose metabolism in the temporal and parietal regions [59]. These correlation results showed that the RMTG is involved in a variety of cognitive processes and further explained the rationale for the involvement of this area. Although the correlation between RMTG metabolism and Aβ deposition did not reach a significant level, it echoes previous reports showing that the reduction in glucose metabolism in AD-sensitive areas is not directly related to Aβ deposition [60, 61], and other evidences suggesting that the elevated brain Aβ deposition alone is probably insufficient to produce neuronal damage and cognitive changes [62, 63]; the correlation between brain Aβ deposition and metabolism is likely to be mediated by neurofibrillary tangles with a temporal delay [60]; however, this was not proven in this study. Based on the above, we thought that the hypometabolism of RMTG in SCD is also reliable in the practical sense, and it may indicate the initiation of nerve injury, and the deterioration of cognitive function in the future.

Several limitations of this study should be addressed. First, SCD is a heterogeneous state and it is affected by the cultural background [14, 15]. Previous studies on its metabolism were inconsistent [12, 13, 30, 31, 33–35], and our results led to new conclusions. Therefore, it should be noted that the participants in our research were all Chinese community-sourced, female-dominated, and comparatively young, whether the hypometabolism of RMTG is a common or unique feature of this population needs to be further confirmed. Second, the amyloid information was not available for the full dataset, and not all the SCD subjects were AD sourced. Third, the small sample number limited the statistical power of our data. We tried to overcome this issue by enrolling participants from another subcenter, but the requirement for FDG-PET data greatly limited the quantity of potential participants in the SILCODE project. Fourth, our study is cross-sectional, and long-term longitudinal follow-up data can further support our conjecture, which is our future research direction. Fifth, we calculated the metabolism with the whole cerebral cortex as reference regions, and choosing different reference areas may affect the results. Sixth, the metabolic difference of MTG was only on the right side, but the current evidence is insufficient to provide a clear explanation for this, and we will continue to explore cerebral functional laterality in future studies. Considering the shortcomings of our research and the limitations in this field, multicenter collaboration to include more confounding factor-matched and pathology-identified participants is needed in the future.

## Conclusion

This dual-center study of a Han population found that the hypometabolism of RMTG could be a potential glucose metabolic biomarker for SCD, the regional metabolism was gradually decreased across the cognitive continuum and showed significant correlations with the degree of subjective complaints, depression, and delayed memory abilities. Furthermore, we suppose that the decreased metabolism of RMTG in the SCD stage may indicate future deterioration of cognitive function, and the specificity of a SCD diagnosis could be increased by detecting RMTG metabolism.

## Supplementary Information

**Additional file 1: Supplementary Table 1.** The ROI-based brain regions with significant differences between SCD and NC by 10 times repeated cross-validations. **Supplementary Table 2.** AUCs of the ROC curves. **Supplementary Figure 1.** Flowchart shows selection of subjects. **Supplementary Figure 2.** The single-subject RMTG SUVR maps of NC, SCD, aMCI, ADD, and DLB individuals from [18F] FDG-PET scans. **Supplementary Figure 3.** ROC curves.

## Data Availability

The dataset generated and analyzed in the current study is available from the corresponding author on reasonable request.

## Abbreviations

AD: Alzheimer’s disease
SCD: Subjective cognitive decline
NC: Normal control
Aβ: β-amyloid
NIA-AA: National Institute on Aging-Alzheimer’s Association
FDG: Fluoro-2-deoxyglucose
PET: Positron emission tomography
ROIs: Regions of interest
PCC: Posterior cingulate cortex
PCUN: Precuneus
SILCODE: Sino Longitudinal Study on Cognitive Decline
aMCI: Amnestic mild cognitive impairment
ADD: Alzheimer’s disease dementia
DLB: Dementia with Lewy body
MRI: Magnetic resonance imaging
AV45: Florbetapir F-18
APOE: Apolipoprotein E
MMSE: Mini-Mental State Examination
MoCA-B: Montreal Cognitive Assessment-Basic
SCD-9: Subjective Cognitive Decline-9
AVLT-N5: Auditory verbal learning test-long delayed memory
AVLT-N7: Auditory verbal learning test-recognition
HAMD: Hamilton Depression
HAMA: Hamilton Anxiety
FOV: Field of view
TR: Repetition time
TE: Echo time
GM: Gray matter
PVE: Partial volume effect
MNI: Montreal Neurological Institute
SUVR: Standardized uptake value ratio
AAL: Anatomical Automatic Labeling
ANOVA: Analysis of variance
GRF: Gaussian random field
ROC: Receiver operating characteristic
AUCs: Areas under curves
CI: Confidence of interval
RMTG: Right middle temporal gyrus
