# Supplementary material for: Glucose metabolism in the right middle temporal gyrus could be a potential biomarker for subjective cognitive decline: a study of a Han population
Source: Alzheimers Res Ther. 2021 Apr 7;13:74. doi: 10.1186/s13195-021-00811-w (PMC8028241; doi:10.1186/s13195-021-00811-w)
Supplement: Supplementary file 1 — Additional file 1: Supplementary Table 1. The ROI-based brain regions with significant differences between SCD and NC by 10 times repeated cross-validations. Supplementary Table 2. AUCs of the ROC curves. Supplementary Figure 1. Flowchart shows selection of subjects. Supplementary Figure 2. The single-subject RMTG SUVR maps of NC, SCD, aMCI, ADD, and DLB individuals from [18F] FDG-PET scans. Supplementary Figure 3. ROC curves. [file 13195_2021_811_MOESM1_ESM.docx]

**Supplementary Table 1.** The ROI-based brain regions with significant differences between SCD and NC by 10 times repeated cross-validations

| Repeat order | The ROIs with significant differences  (ROI number of AAL template) | |
| --- | --- | --- |
|  | First fold | Second fold |
| 1 | 5, 6, 9, 10, 37, 38, 43, 45, 46, 49, 50, 51, 53, 56, 59, 61, 64, 82, 86 | 7, 11, 24 , 52, 56, 64, 77, 78, 82, 86 |
| 2 | 5, 9, 10, 17, 43, 45, 46, 49, 55, 56, 59, 61, 65, 71, 82, 86, 89, 90 | 23, 64, 86 |
| 3 | 3, 5, 21, 40, 41, 43, 44, 45, 46, 49, 56, 77, 79, 86, 89 | 17, 64, 82, 86 |
| 4 | 17, 22, 31, 32, 34, 45, 52, 54, 64, 66, 78, 82, 86 | 5, 6, 11, 21, 22, 27, 40, 41, 42, 43, 45, 46, 49, 55, 56, 61, 77, 86 |
| 5 | 1, 5, 9, 43, 45, 46, 49, 57, 59, 61, 64, 65, 82, 86 | 17, 26, 31, 32, 45, 55, 56, 64, 71, 73, 86 |
| 6 | 5, 11, 43, 45, 52, 64, 86 | 29, 30, 43, 45, 46, 49, 56, 58, 64, 71, 72, 77, 78, 82, 86, 90 |
| 7 | 6, 11, 18,43, 46, 52, 56, 77, 78 , 82, 86 | 5, 17, 45, 49, 64, 86, 89 |
| 8 | 5, 6, 10, 45, 46, 49, 50, 64, 86, 89 | 1, 52, 56, 57, 61, 82, 86 |
| 9 | 5, 9, 17, 43, 62, 64, 74, 75, 76, 78, 82, 86 | 18, 35, 36, 38, 43, 45, 46, 49, 56, 59, 60, 61, 76, 82, 86, 88, 90 |
| 10 | 5, 6, 9, 10, 17, 32, 45, 46, 49, 50, 64, 77, 86 | 37, 38, 39, 40, 42, 43, 61, 66, 71, 72, 82, 86 |

Abbreviations: SCD, subjective cognitive decline; NC, normal control; ROI, region of interest; AAL, anatomical automatic labeling.

**Supplementary Table 2.** AUCs of the ROC curves

| Metabolism | AUCs (95% CI) | | | | | |
| --- | --- | --- | --- | --- | --- | --- |
|  | SCD1 vs. NC1 | SCD2 vs. NC2 | SCD vs. NC | aMCI vs. NC2 | ADD vs. NC2 | DLB vs. NC2 |
| RMTG | 0.638 (0.481–0.788) | 0.717 (0.577–0.857) | 0.682 (0.586–0.780) | 0.959 (0.914–1.000) | 0.997 (0.989–1.000) | 1.000 (1.000–1.000) |
| PCC | 0.599 (0.453–0.749) | 0.604 (0.465–0.754) | 0.534 (0.425–0.635) | 0.935 (0.879–0.996) | 0.899 (0.834–0.973) | 0.920 (0.856–0.987) |
| PCUN | 0.499 (0.349–0.655) | 0.562 (0.412–0.713) | 0.540 (0.433–0.642) | 0.720 (0.600–0.842) | 0.617 (0.474–0.763) | 0.741 (0.598–0.879) |

Abbreviations: AUCs, areas under curve; CI, confidence interval; ROC, receiver operating characteristic; SCD, subjective cognitive decline; NC, normal control; aMCI, amnestic mild cognitive impairment; ADD, AD-dementia; DLB, dementia with Lewy body; RMTG, right middle temporal gyrus; PCC, posterior cingulate cortex; PCUN, precuneus.


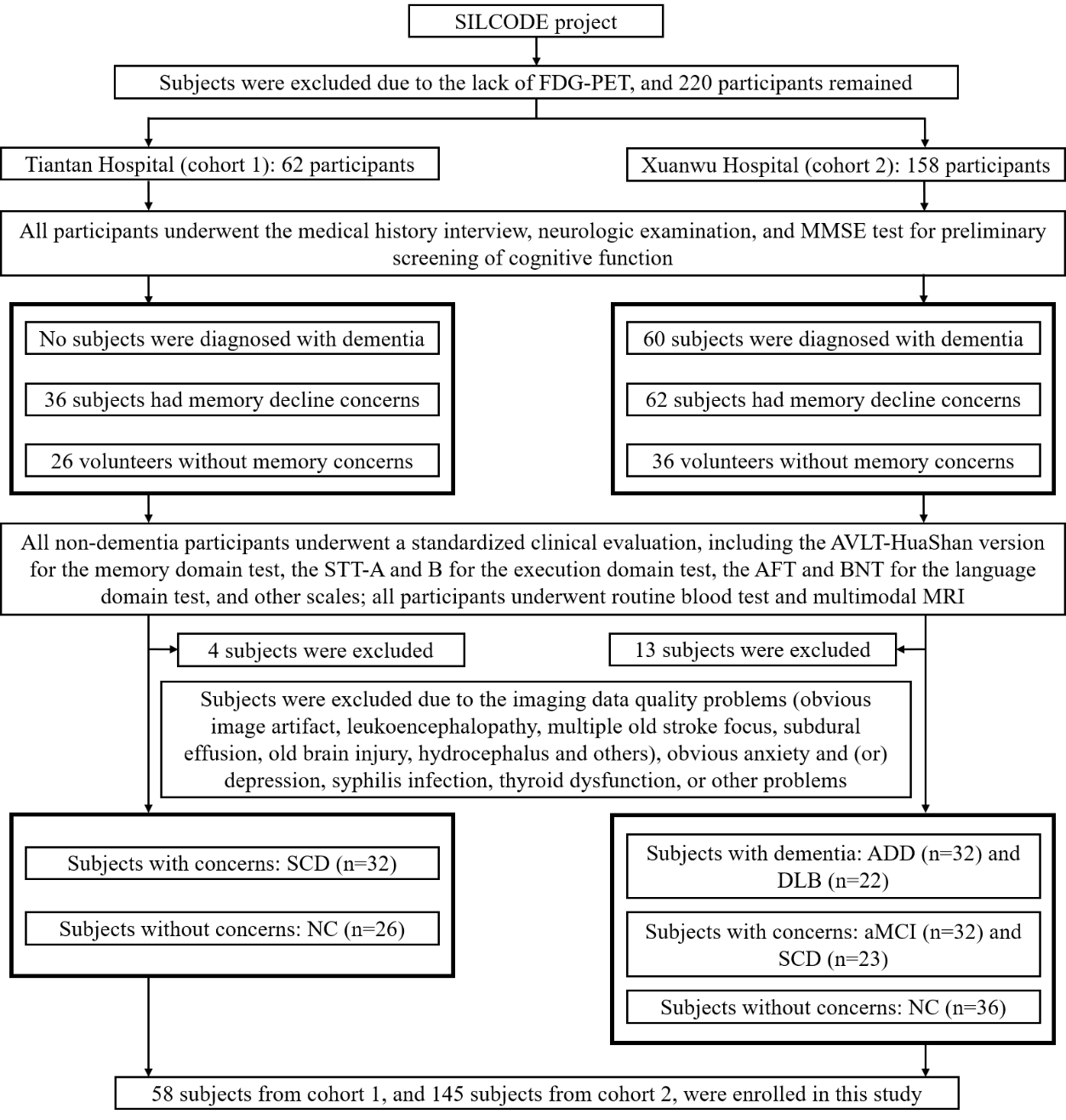
**Supplementary Figure 1.** Flowchart shows selection of subjects.

Abbreviations: SILCODE, Sino Longitudinal Study on Cognitive Decline; FDG, [18F]fluoro-2-deoxyglucose; PET, positron emission tomography; MMSE, mini-mental state examination; AVLT, auditory verbal learning test; STT, shape trails test; AFT, animal fluency test; BNT, 30-item Boston naming test; MRI, magnetic resonance imaging; SCD, subjective cognitive decline; NC, normal control; ADD, Alzheimer’s disease dementia; DLB, dementia with Lewy body; aMCI, amnestic mild cognitive impairment.


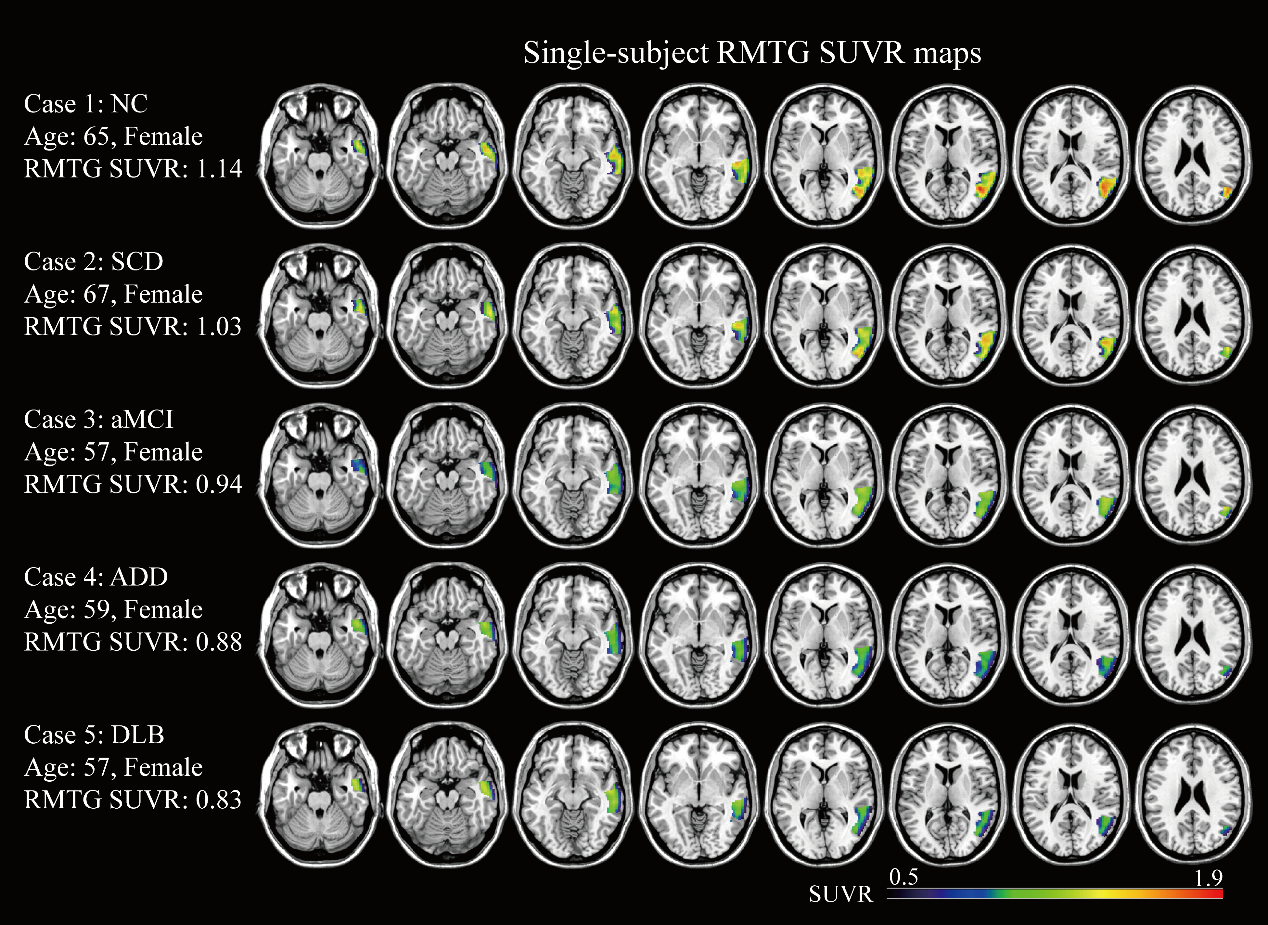


**Supplementary Figure 2.** The single-subject RMTG SUVR maps of NC, SCD, aMCI, ADD, and DLB individuals from [18F] FDG-PET scans. The threshold of the SUVR value was set range from 0.5 to 1.9. Cool colors represent hypometabolism and hot colors represent hypermetabolism. All selected individuals were female. The RMTG SUVR of a 65-year-old NC individual was 1.14, of a 67-year-old SCD individual was 1.03, of a 57-year-old aMCI individual was 0.94, of a 59-year-old ADD individual was 0.88, and of a 57-year-old DLB individual was 0.83. Notably, although the ages of aMCI, ADD and DLB subjects were younger than the NC and SCD subjects, their metabolisms of RMTG were still lower. This indicate that the decrease of RMTG glucose metabolism might due to the cognitive changes, instead of the influences of aging.

Abbreviations: RMTG, right middle temporal gyrus; SUVR, standardized uptake value ratio; NC, normal control; SCD, subjective cognitive decline; aMCI, amnestic mild cognitive impairment; ADD, AD-dementia; DLB, dementia with Lewy body; FDG- PET, fluoro-2-deoxyglucose positron emission tomography.


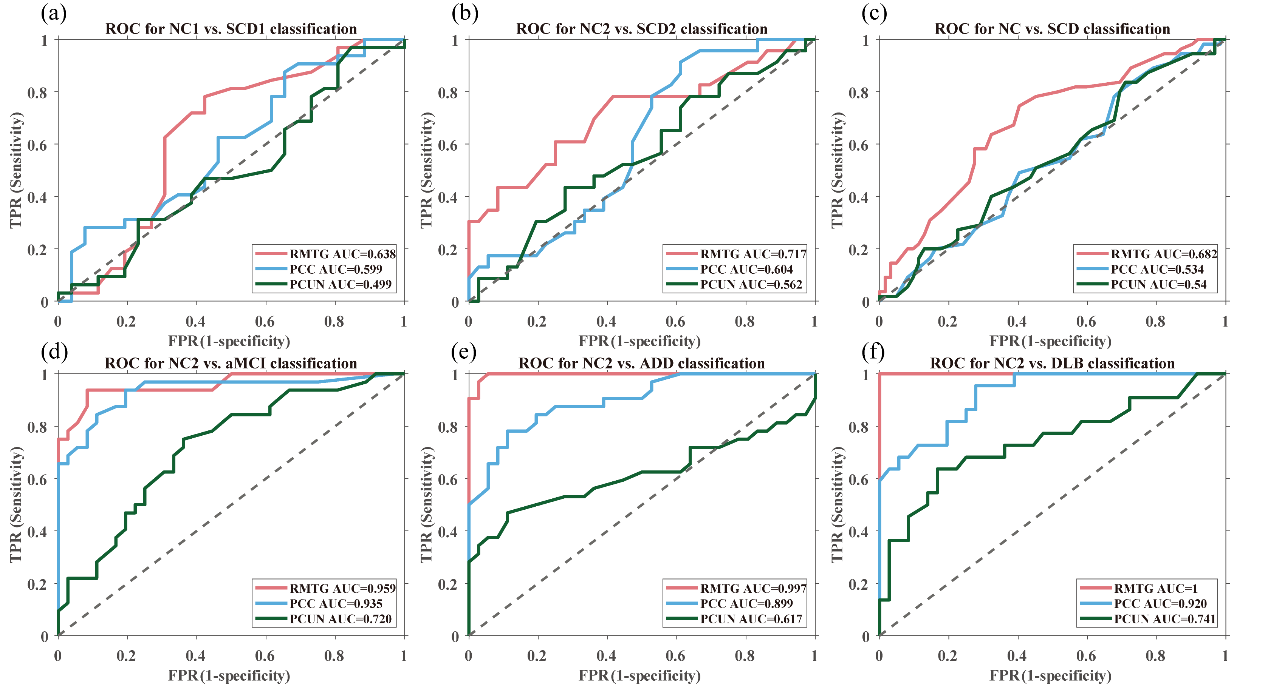


**Supplementary Figure 3.** ROC curves. The NC1 vs. SCD1 classification was shown in (a), NC2 vs. SCD2 classification in (b), NC vs. SCD classification in (c), NC2 vs. aMCI classification in (d), NC2 vs. ADD classification in (e), NC2 vs. DLB classification in (f). In addition to using the metabolism of RMTG, we also used the metabolism of PCC and PCUN to differentiate groups.

Abbreviations: ROC, receiver operating characteristic; NC, normal control; SCD, subjective cognitive decline; aMCI, amnestic mild cognitive impairment; ADD, AD-dementia; DLB, dementia with Lewy body; RMTG, right middle temporal gyrus; PCC, posterior cingulate cortex; PCUN, precuneus; SUVR, standardized uptake value ratio; AUC, areas under curve.
